# Healable Supracolloidal Nanocomposite Water-Borne Coatings

**DOI:** 10.1021/acsapm.4c00946

**Published:** 2024-07-22

**Authors:** Siyu Li, Leendert G. J. van der Ven, Santiago J. Garcia, A. Catarina C. Esteves

**Affiliations:** † Laboratory of Physical Chemistry, Department of Chemical Engineering and Chemistry, Eindhoven University of Technology, P.O. Box 513, Eindhoven 5600 MB, The Netherlands; ‡ Aerospace Structures and Materials Department, Faculty of Aerospace Engineering, Delft University of Technology, Kluyverweg1, Delft 2629 HS, The Netherlands; § Institute for Complex Molecular Systems (ICMS), Eindhoven University of Technology, P.O. Box 513, Eindhoven 5600 MB, The Netherlands

**Keywords:** coatings, self-healing, nanocomposites, supracolloids, water-borne

## Abstract

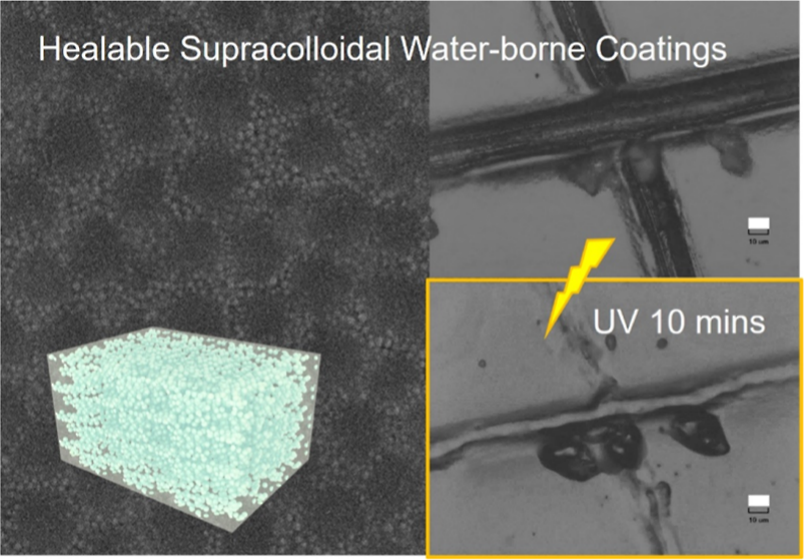

Water-borne coatings often contain nanofillers to enhance their mechanical or optical properties. The aggregation of these fillers may, however, lead to undesired effects such as brittle and opaque coatings, reducing their performance and lifetime. By controlling the distribution and structural arrangement of the nanofillers in the coatings and inserting reversible chemical bonds, both the elasticity and strength of the coatings may be effectively improved, while healing properties, via the reversible chemistry, extend the coating’s lifetime. Aqueous dispersions of polymer-core/silica-corona supracolloidal particles were used to prepare water-borne coatings. Polymer and silica nanoparticles were prefunctionalized with thiol/disulfide groups during the supracolloid assembly. Disulfide bridges were further established between a cross-linker and the supracolloids during drying and coating formation. The supracolloidal nanocomposite coatings were submitted to intentional (physical) damages, i.e., blunt and sharp surface scratches or cut through into two pieces, and subsequently UV irradiated to induce the recovery of the damage(s). The viscoelasticity and healing properties of the coatings were examined by dynamic, static, and surface mechanical analyses. The nanocomposite coatings showed a great extent of interfacial restoration of cut damage and surface scratches. The healing properties are strongly related to the coating’s viscoelasticity and interfacial (re)activation of the disulfide bridges. Nanocomposite coatings with silica concentrations below their critical volume fraction show higher in situ healing efficiency, as compared to coatings with higher silica concentration. This work provides insights into the control of nanofillers distribution in water-borne coatings and strategies to increase the coating lifetime via mechanical damage recovery.

## Introduction

Water-borne dispersions and coatings have been extensively studied in the past 20 years as more environmentally friendly and sustainable alternatives to organic solvent-borne systems.^1,2^ Water-dispersible colloidal systems, i.e., latexes, are the largest family among the current water-borne technologies used in, among others, indoors and wood coating applications. Due to the nature and type of the polymers dispersed in such aqueous systems, water-borne coatings typically contain high loads of fillers, pigments, or other additives to enhance their thermo-mechanical properties and overall performance. However, this relatively high load of particles, together with the constraints of the film formation process in water-borne systems, can cause undesired effects such as agglomeration of the particles, which ultimately result in inferior, e.g., mechanical or optical, properties. Therefore, several strategies have been proposed to increase the mechanical endurance of water-borne coatings. On one hand, nanocomposite strategies have been proposed to increase the resistance to mechanical damage.^3−5^ On the other hand, a less explored route in water-borne coatings proposes mechanical damage management through healing. The use of (self-)healing mechanisms intends to implement repairing strategies to recover functionalities (mechanical strength, optical, or barrier properties) lost during damage in order to maintain a high performance over an extended lifetime. When coatings are only slightly locally damaged, the implementation of healing mechanisms can also reduce maintenance costs, which ends up increasing the overall sustainability of the coated products.

**Scheme 1:**
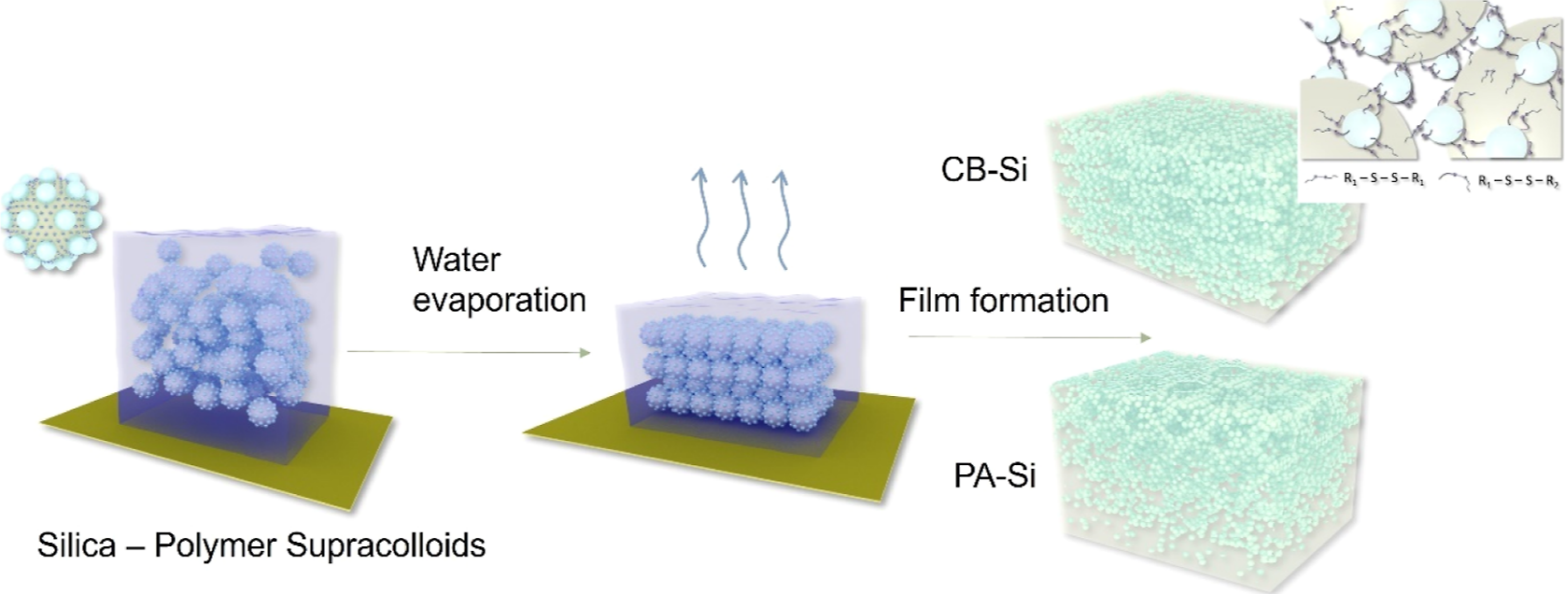
Schematic of the Preparation of the Nanocomposite Coatings: PA-Si Coatings, with the Silica Nanoparticles Physically Adsorbed on the Polymer Surfaces, and CB-Si Coatings with Silica and Polymer Particles Covalently Bound via Dynamic Disulfide Bonds (Inset on the Right)

From the perspective of using nanocomposites, fillers such as carbon nanotubes, silica nanoparticles, titanium dioxide, clay, and calcium carbonate have been embedded into polymer binders for different coating application requirements.^4,6−10^ In these cases, the distribution of the nanofillers and the filler network structure are two critical parameters to effectively enhance the desired coating properties. A well-defined network structure throughout the coating can, for instance, improve oxygen permeability, mechanical, thermal, optical, and conductivity properties.^11−13^ The cross-linking between fillers and polymers has also been reported to improve the dispersion of the nanofillers, as well as the interfacial compatibility and adhesion between fillers and polymer binders.^14,15^

Considering the introduction of the self-healing function into coatings, intrinsic healing approaches are of key interest as they do not rely on external agents to regain the coating properties.^16,17^ Various chemical functionalities can be used to achieve intrinsic healing in polymer coatings at the molecular level. One of the approaches is to incorporate reversible covalent bonds,^18^ using disulfide bridges^19,20^ trithiocarbonate groups,^21^ Diels–Alder adducts,^22−24^ or Si–O–Si reversible networks, in basic conditions.^25^ In general, a healing capability provided by reversible bonds incorporated into a network can reconstruct the damaged material in a short time frame upon relatively simple triggers or stimuli (e.g., temperature). Healing based on the diffusion of thermoplastic polymer networks, above *T*_g_, often requires a long(er) time and exposure to heat.^16,26−28^ An alternative to reversible covalent bonds is the use of supramolecular interactions,^29^ such as hydrogen bonds,^30^ π–π stacking,^31^ electrostatic interactions in polyelectrolytes,^32,33^ and various host–guest interactions.^34−36^ Despite the options available, choosing suitable healing mechanisms and chemistries for water-borne coatings remains challenging due to the presence of water as the medium and the requirement to incorporate a large fraction of inorganic particles, i.e., nanofillers or pigments.

Many healable nanocomposites have been reported that end up with improved thermal or mechanical properties. For instance, healing of composites consisting of polymer binders of high *T*_g_(37) combined with gold nanoparticles,^38^ graphene,^39−42^ nanocellulose,^34,43−47^ silica nanoparticles,^48,49^ carbon nanotubes,^50^ boron nitride nanosheets,^51^ titanium dioxide nanoparticles,^52^ or calcium carbonate^53^ have been investigated. Besides, a few studies reported the use of modified nanofillers with reversible chemical bonds, taking advantage of the large specific surface area of the nanoparticles to achieve improved healing results.^54−56^ However, the use of reversible bonds between inorganic (nano)fillers and polymer colloids has not yet been studied in aqueous dispersions and water-borne coatings.

Previously, we reported water-borne coatings obtained from aqueous dispersions of polymer(core)–silica(corona) supracolloidal particles.^57^ The use of covalently bonded polymer–silica supracolloids with a partially covered corona, i.e., strawberry configuration,^57^ prevented the destabilization of the nanoparticles, namely, during film formation, and resulted in well-arranged 3D silica (nano)network structures throughout the coating (Scheme 1).^58^ The films were cross-linked through the establishment of disulfide bonds between the polymer–silica particles within supracolloids, silica–silica particles on the supracolloid coronas, and polymer–polymer particles across the supracolloid interface, upon polymer coalescence and film formation.^58^ We also investigated the effect of the presence of poly(ethylene oxide) chains on the assembly of the supracolloids with the well-defined strawberry configuration^59^ and the fine-tuning of the system to avoid extensive (surface) segregation of the silica nanofillers upon film formation. The storage moduli and the water resistance of the coatings obtained from covalently bonded supracolloids were considerably improved, when compared to those of coatings made from supracolloids stabilized by physical adsorption only.^58^ How this (property) enhancement was affected by the overall silica nanoparticles concentration and the presence of reversible disulfide bonds (which provided the cross-linked network) was, however, not addressed in our previous studies. The possible exchange of sulfur–sulfur bonds has been long since reported,^60^ and disulfide bonds have been substantially applied for reversible chemistry for polymeric (self-)healing materials, e.g., epoxy thermosets^20,61^ and polyurethanes.^62−65^ This perspective was, however, not investigated in our previously reported water-borne colloidal system.

In this work, a strategy to simultaneously enhance the mechanical resistance and healing capability of water-borne coatings is described, using preformed supracolloidal particles and reversible bonds for the cross-linking of the nanocomposite polymer coatings. The coatings were investigated by different thermo-mechanical methods, which provided a comprehensive understanding of the silica networks formed and of their viscoelastic properties. Furthermore, the healing properties of the system, via the reshuffling of disulfide bonds, were also investigated as a function of the silica nanoparticle concentration and related to the viscoelastic properties of the water-borne nanocomposite coatings.

## Materials and Methods

### Materials

Butyl acrylate (99%, BA), methyl methacrylate (99%, MMA), and ethylene glycol dicyclopentenyl ether methacrylate (90%, DCPMA) were purchased from Sigma-Aldrich and purified via a basic alumina column (Honeywell, Brackman I) to remove inhibitors before polymerization. 2,2′-(Ethylenedioxy)diethanethiol (95%, EDT), 2-hydroxy-4′-(2-hydroxyethoxy)-2-methylpropiophenone (98%, HMPP), 2-amino-2-methyl-1,3-propanediol (99%, AMPD), citric acid (99%), poly(ethylene glycol) methyl ether methacrylate (PEGMA) solution (Mn ∼ 2000 g/mol, 50 wt %), Triton X-405 solution (Mn ∼ 1967 g/mol, 70 wt %), Zeolite 13×, and the initiator 2,2′-azobis[2-(2-imidazolin-2-yl) propane] dihydrochloride (98%, AIBA) were used as received from Acros Organics. Levasil silica aqueous colloidal dispersion (40 wt %, thiol functionalized) was kindly provided by Nouryon (Sweden) and further purified by dialysis or diafiltration as described in ref (57). The AMPD-citric acid aqueous solution was used as a buffer to adjust the pH and dilute the polymer and silica dispersions. Deionized water (pH 6.5) filtered with an Elix Reference water purification system was used for all experiments.

### Supracolloidal Dispersions Preparation

The preparation of the supracolloidal dispersions was performed as described in ref (57) and the coatings were prepared as described in ref (58). The supracolloids used for this particular study were prepared as follows: the polymer particles (named as L.33, where 0.33 stands for the theoretical PEGMA chain number at polymer particle surface per nm^2^) were obtained by semicontinuous emulsion polymerization using equiponderant quantities of two main monomers, i.e., MMA and BA. Triton X-405 and PEGMA were used as stabilizers for polymerization and as assembly promotors. A comonomer DCPMA was added in the last step of the semicontinuous emulsion polymerization to introduce vinyl group functionalities at the polymer surface, which can undergo thiol/ene click reactions.^57^ These vinyl groups at L.33 were converted afterward into thiol groups by a prereaction with EDT, which also acts as a cross-linker during the film formation. The thiol/ene reaction was carried out under UV irradiation.^57^ No clear variation in the hydrodynamic diameter of the polymer core (D ≈ 255 nm) was detected before and after the reaction with EDT. After (EDT) thiol functionalization, the polymer particles were named EDT-L.33.

The supracolloidal dispersions were prepared by simply blending individual (thiol-)polymer and (thiol/disulfide)-silica colloidal aqueous dispersions. The silica corona configuration was precisely controlled by the assembly conditions, as reported in our previous work.^57^ At the selected dispersions concentrations and mixing conditions, silica nanoparticles partially covered the polymer surface (in a strawberry configuration), while the fraction of free-standing silica nanoparticles in the dispersion remains negligible.^57^ The corona of the strawberry supracolloids consists of silica nanoparticles with a diameter of about 27 nm (estimated from transmission electron microscopy images) functionalized with thiol groups, which were oxidized to disulfides (−S–S−) upon air exposure.^57^

### Free-Standing Supracolloidal Nanocomposite Coatings Preparation

Supracolloidal dispersions with 12 wt % of supracolloids, prepared as previously described,^57^ were cast as polymer films on Teflon substrates using an applicator with a 250 μm gap and dried in a glass curing box with a stable airflow at ambient temperature (22 ± 2 °C) and 5 ± 2% humidity conditions for 6 h. The thiol groups present were also converted to disulfides during this step. The films were easily peeled from the substrate by hand and had a thickness of ≈80–120 μm, as measured by a digital micrometer (54–815 series, Fowler Xtra-value). The prepared coatings and their composition and nomenclature are given in Table 1.

**Table 1:** Composition of the Nanocomposite Coatings—Silica Nanoparticles Mass Percentage (*C*_silica_) and Volume Fraction (ϕ_silica_) and EDT [Crosslinker] Mass Fraction (*C*_EDT_)

coatings	*C*_silica_ (wt %)	ϕ_silica_	*C*_EDT_ (wt %)
aL.33	0		
aEDT-L.33	0		2.9
bCB-Si.*xx*	15	0.075	2.8
	20	0.1	2.6
	25	0.125	2.5
cPA-Si.*yy*	15	0.075	
	20	0.1	
	25	0.125	
	40	20	

a Coatings with polymer particles (L.33) only.

b Covalently bound supracolloids.

c Physically adsorbed supracolloids.

### Thermo-Mechanical Properties

The glass transition temperature (*T*_g_) of the polymer films was determined with differential scanning calorimetry (TA Q2000 instrument). The samples were measured using ∼15 mg of dried polymer films and two consecutive heating–cooling cycles in the temperature range from −20 to 80 °C at 20 °C/min. *T*_g_ was estimated from the second heating cycle by the half-heat capacity method using TA Universal Analysis software. The *T*_g_ of the L.33 polymer film was 13.3 °C, and EDT had a minimal effect on *T*_g_ of EDT-L.33, which was 13.0 °C (Figure S8). As for the nanocomposite coatings, the *T*_g_ values were between 17 and 22 °C, depending on the silica concentration (Figure S9).

Since the coatings prepared exhibited very different silica distributions throughout the bulk, their thermo-mechanical properties were evaluated by two methods. Before any measurements, the films were put in an aging box at 70 °C with a stable N_2_ flow for 48 h to obtain stable films.

#### Rheometer with a Plate-To-Plate Configuration

Temperature sweep tests of the storage modulus, loss modulus, and loss factor were performed by a rheometer (Physica MCR 301, Anton Paar) equipped with an H-ETD400 temperature control device, using a plate-to-plate geometry, where the plate diameter is 7.946 mm (Anton Paar Datasheet). The measurements were conducted by the application of a constant normal force of 0.25 N to the film with an angular frequency of 1 rad/s and a strain of 0.05%. The strains were chosen in the linear elastic region of the supracolloidal films. Before each measurement, the films were relaxed under 0.25 N axial force for 3 min at 22 ± 0.5 °C to reach an optimal contact between measurement plates and the sample. Then, an increasing temperature profile was applied, in which the temperature was increased from 22 to 90 °C for 140 min, and 840 data points were obtained with an interval of 10 s. Three measurements were conducted for each film prepared in different batches. The average results of three measurements were further averaged for every five points to reduce the noise caused by the limited resolution of the rheometer.

#### Dynamic Mechanical Thermal Analyses

Mechanical tests of all free-standing films were also performed on a DMA Q850 (TA Instruments) in the tension mode, with a film tension fixture at 23 ± 0.5 °C. The tensile test specimens were cut from the annealed coatings into pieces of about 2.7 mm width using a film cutter with two parallel razor blades. The precise dimensions of each sample were measured three times, and the average value was taken. A preload force of 0.005 N and 1 min interval time were used to stabilize the sample. The entry length between the two film clamps was controlled between 4.6 and 5.0 mm. The drawing velocity was constant at 1 mm/min, and the maximum strain of all samples was controlled up to 400% as the limit displacement of the instrument was reached. The Young’s modulus *E* of each sample was determined at strain = 5% using equation 1.
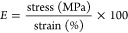
1

### Nontriggered (Viscoelastic) Recovery Assessment

A micro scratch tester/indenter (MST, CSM Instruments) was used to create well-defined surface scratches. The instrument was equipped with a 100 μm radius sphere-conical diamond intender tip (Rockwell). The scratch length (3 mm) and scratching speed (5 mm/min) were kept constant in all experiments. Four scratches with different loads (0.2 0.5, 1, and 1.5 N) were created for each specimen. The scratching procedure also contained prescan and postscan steps at 0.03 N load that were used to probe the surface topology before and (typically 30 min) after inflicting the scratch. The penetration depth of the tip was obtained directly during scratching. The elastic recovery was obtained from the postscan measurement (residual depth measured during postscan minus depth during the scratching process). The regressive scratch gap depth (scratch healing) was measured by a 3D Laser Scanning Microscope (VK-X 3000, Keyence) using a 10× objective. A zero value indicates the initial coating surface and, hence, corresponds to a fully recovered gap. For one image, the average regressive gap depth of 6 different positions (same positions for all images) was measured sequentially. The regressive gap depth of each coating was an average result of sequential measurements at 12 different positions from two different coating batches.

### Triggered (UV Irradiation) Recovery Assessment

To evaluate the damage healing upon UV triggering, notch cuts were made by hand using a triangular scalpel blade (Swann-Morton). Then, the damaged coatings were subsequently put under a Mercury spot UV curing lamp (S1000, OmniCure, 320–500 nm) with EXFO fiber light guides. The irradiation distance to the light lenses was vertically controlled at 50 mm (131 mW/cm^2^). After 10 min of irradiation, the samples were immediately checked by an optical microscope (BX51, Olympus).

The ability of the coatings to recover surface damage upon UV irradiation was also examined by measuring the regressive gap depth of a scratch as described above. To this aim, the scratch gap depth was measured before and after 10 min of UV irradiation using a confocal microscope.

**Figure 1:**
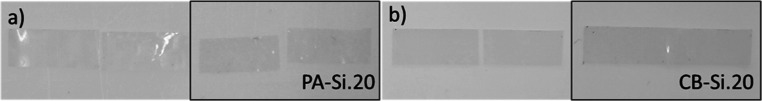
Photographs of the cut/rejoined coating specimens: (a) PA-Si.20 and (b) CB-Si.20 nanocomposite films (tensile test strips). The nomenclature of coatings is explained in Table 1. The specimens after UV irradiation are indicated in purple frames (right images of Figure 1a,b).

**Figure 2:**
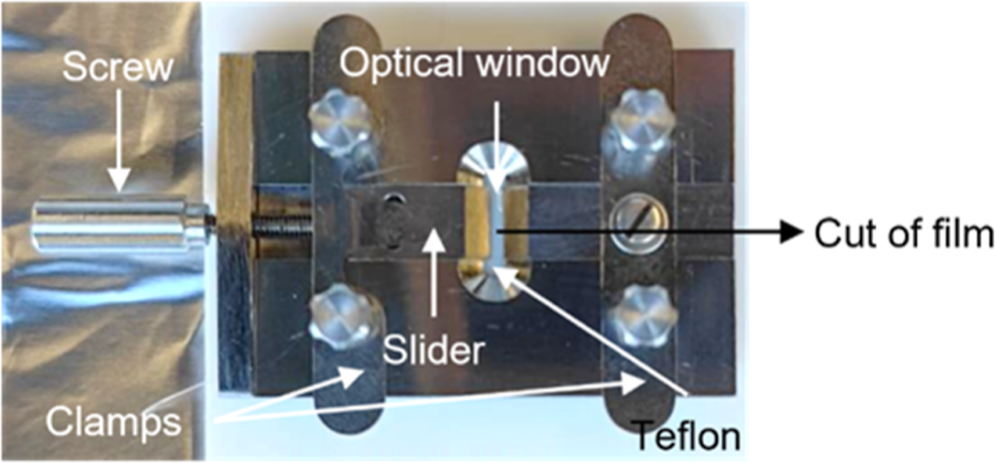
Digital photographs of the setup for the cut-joined healing experiments: clamps and clamped coating tensile test strip. The optical window of about 2 × 8 mm^2^ was used to bring the two parts in contact and have good alignment during the UV irradiation.

To test the healing potential of fully cut films, a cross-cut was made in the middle of predefined rectangular tensile test strips (20 × 5.4 mm^2^), as shown in Figure 1. The two pieces of the cut coating were transferred into a homemade film clamp apparatus, as shown in Figure 2.

As shown in Figure 2, the film clamps have a Teflon plate as the low-friction substrate to place the coatings and align them well. The left side of the film clamp was designed as a slider that was able to move the left piece of the coating with a screw. The left piece of the specimen can contact the right piece, which was previously fixed in the right clamp. Hence, only the cross-sections of the two pieces were in contact without overlapping each other. The interface between two pieces of the specimen was placed in the middle of the optical window (on the top of the Teflon). The optical window was approximately 2 mm wide so that the UV irradiation can be effectively applied to the damaged region without heavily interfering with the whole film. The two film pieces in contact were aligned by the film clamps and photoirradiated for 10 min as well. The distance between the UV lamp and the optical window was approximately 50 mm. The tensile test of rejoined coating specimens was conducted approximately 30 min after the UV irradiation process, unless the stabilizing period was specifically mentioned, e.g., after 20 days for the CB-Si.20 coating.

## Results and Discussion

Polymer–silica supracolloids were prepared by establishing covalent bonds (CBs) between the particles via disulfide bridges.^57^ The supracolloids were used to make nanocomposite coatings, which were investigated regarding their potential healing properties. Table 1 summarizes all coatings prepared at different silica mass percentages (CB-Si.*xx*, where *xx* = 15, 20, or 25 wt %). A disulfide cross-linker EDT was added to all coating formulations. To serve as a reference, coatings prepared with supracolloids formed only by physical adsorption (PA) between silica and polymer particles and to which no disulfide cross-linker was added were also prepared and named as PA-Si.*yy*, where *yy* denotes the silica mass percentage of the supracolloids (*yy* = 15, 20, 25, and 40 wt %).

**Figure 3:**
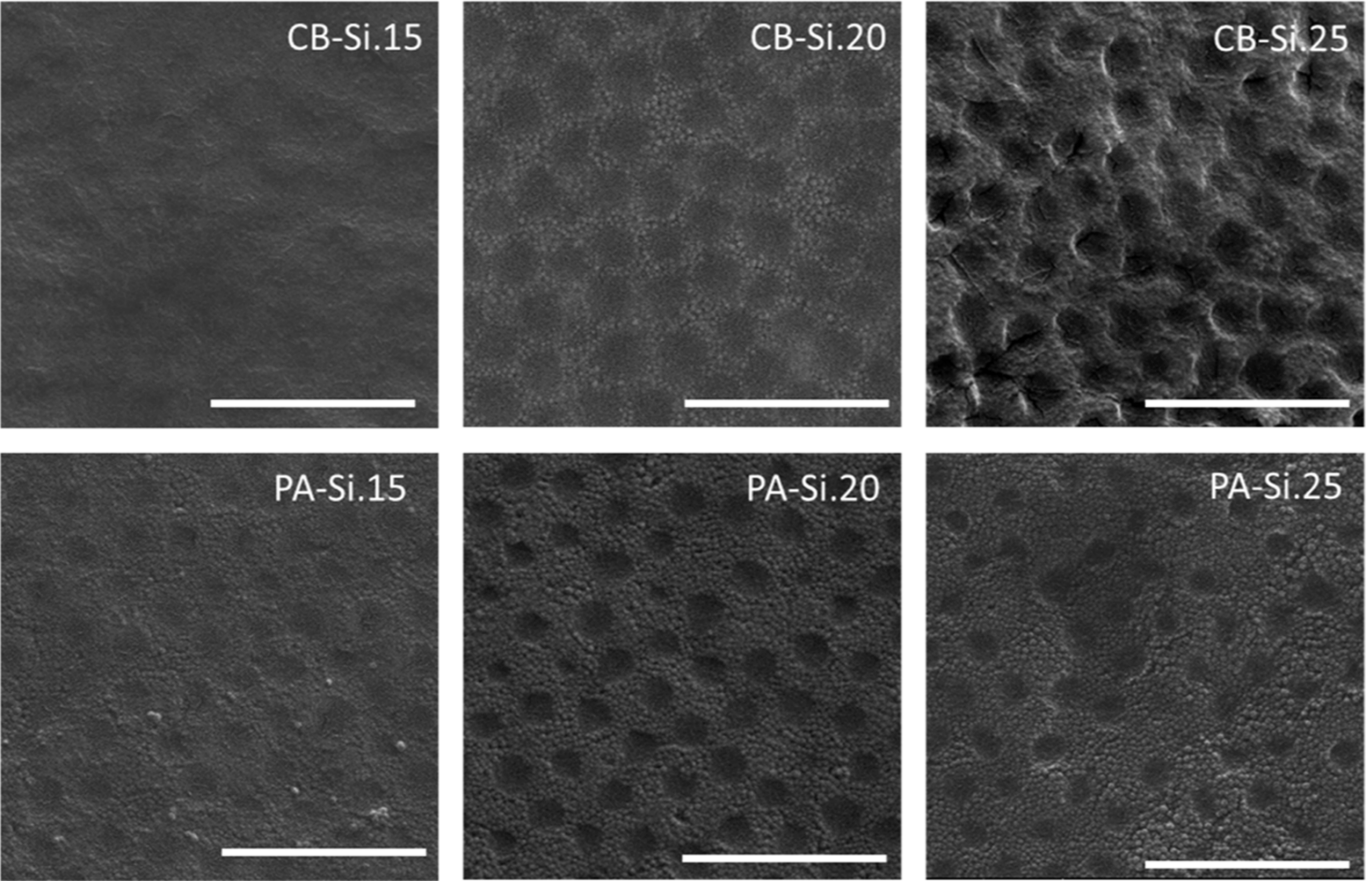
Scanning electron microscopy images of the air–coating interface of CB- and PA-Si nanocomposite coatings (scale bars = 1 μm). Silica segregation is more visible in the PA-Si samples, i.e., higher concentration of *light gray smaller spheres* in certain regions of the micrographs.

The CB-Si coatings prepared are structurally isotropic, i.e., with a continuous 3D silica nanonetwork, while PA-Si coatings have a gradient distribution of silica nanoparticles throughout the depth of the coating, with a high silica concentration at the air–coating interface and a low concentration at the substrate–coating interface, Figure 3. The full characterization of the coating’s morphology has been reported elsewhere.^58^ After annealing the CB-Si coatings at 70 °C for 48 h, ∼ 80% of all thiol groups present were converted to disulfides, as estimated by Raman spectroscopy (Figure S1 and the adjacent text), resulting in cross-linked coatings with reversible disulfide bonds. Such bonds are known to form sulfur radicals by bond cleavage under photoirradiation and undergo bonds exchange within polymer systems. This reshuffling of −S–S– bridges can assist the repair of damages in polymer coatings.^66,67^

The viscoelastic properties of the polymers used here in the nanocomposite coatings are critical for the first step of the healing process–polymer reflow.^68^ Therefore, the mechanical properties of the CB coatings were first investigated and compared to the properties of coatings prepared from polymer particles only (L.33 and EDT-L.33) and physically adsorbed supracolloids (PA). Next, the mechanical properties of CB-Si and PA-Si coatings prepared with different percentages of silica were characterized and related to their healing capability, with respect to recovering from mechanically inflicted damages, i.e., scratches and cut-through.

### Mechanical Properties of Nanocomposite Coatings

**Figure 4:**
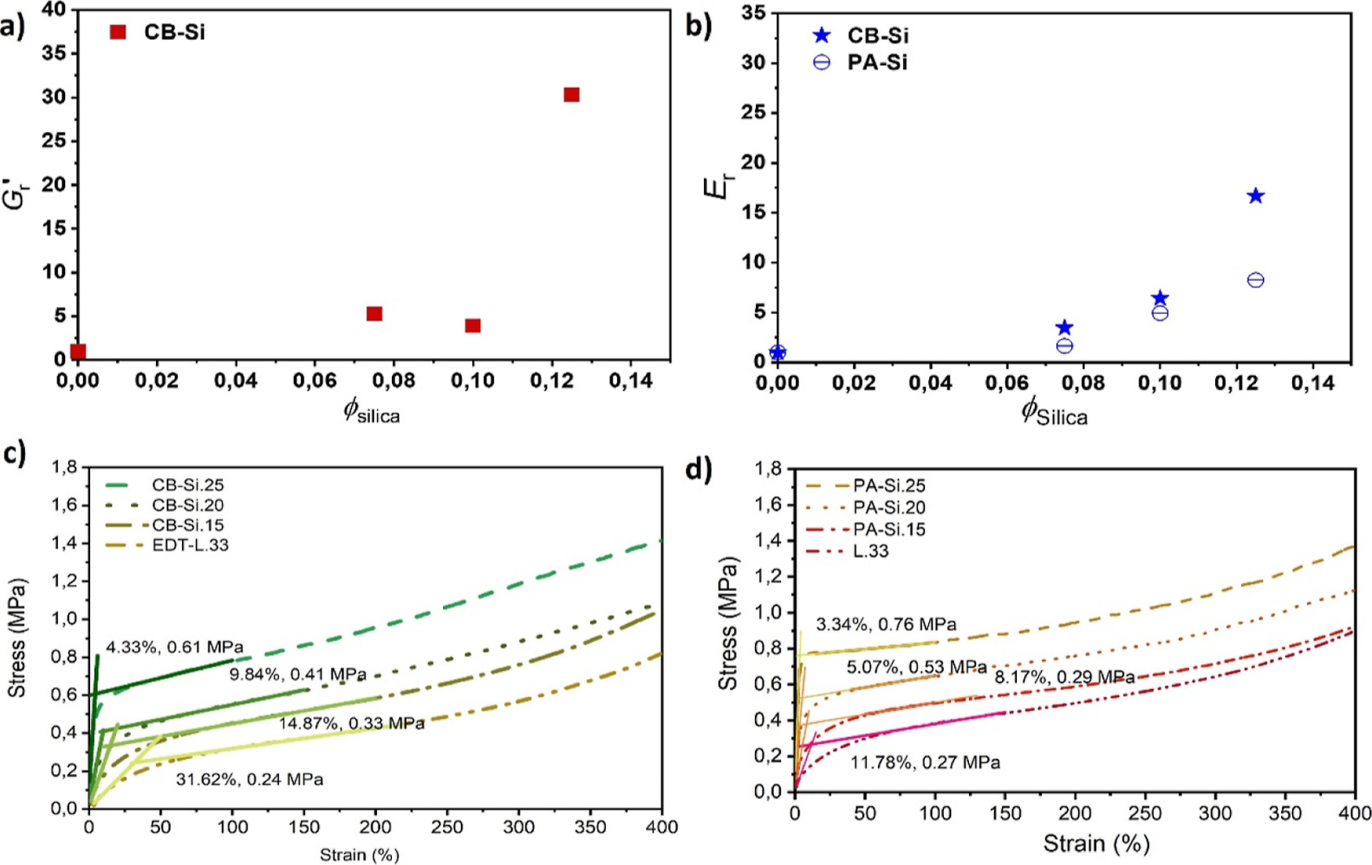
Viscoelastic properties of nanocomposite coatings: (a) relative storage moduli (*G*′_r_) versus ϕ_Si_ of CB-Si at 23 °C, measured at 1 rad/s angular frequency and 0.05% strain. (b) Relative Young’s moduli (*E*_r_) versus ϕ_Si_ of CB-Si and PA-Si measured at 23 °C. (c,d) Stress–strain measured on (c) CB-Si nanocomposite and EDT-L.33 coatings and (d) PA-Si nanocomposite and polymer only (L.33) coatings. The values reported over the curves are the estimated yield stress point and the *E* modulus at the yield point for each represented sample.

The relation between the silica fillers mass fraction and the moduli of the nanocomposite coatings was first evaluated based on the results in Figure 4. The Kerner equation has been often used to describe the relation between the storage modulus *G*′ of a nanocomposite material and the fillers volume fraction ϕ_filler_.^69−72^ However, this equation becomes limited when the volume fraction of the filler exceeds the aggregation concentration (critical volume fraction, ϕ_c_). Lewis and Nielsen^70^ used the critical volume fraction ϕ_c_ to modify the original Kerner equation as follows
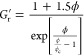
2where *G*′_r_ (= *G*′_nanocomposite_/*G*′_polymer_) is the relative storage modulus of the material and *G*′_polymer_ corresponds to the storage modulus of the respective polymer material alone (without fillers). This equation had the boundary condition of ϕ ≤ ϕ_c_, and ϕ_c_ was estimated from the random packing efficiency of spheres to a value of 0.64. However, the effective volume fraction of fillers in the nanocomposite can be affected by the thin polymer layer adsorbed at the filler’s surfaces. Hence, in another study,^73^ this effect was taken into account, and the effective critical volume fraction ϕ_c,eff_ of the fillers was estimated to a value of 0.30. In the nanocomposite coatings studied here, the relative storage modulus (*G*′_r_) of CB-Si (ϕ_Si_ = 0.125) (Figure 4a) is much higher than what would be estimated with ϕ_c,eff_ = 0.30 and the modified Kerner equation (*estimation and detailed discussion* in Supporting Information, Figure S10).

For polymer coatings comparable to the ones used in this study, a close-packed honeycomb structure of the polymer particles is expected in the deformation stage, as reported by Routh and Russel.^74^ Hence, it is reasonable to assume that in the nanocomposite coatings, each polymer particle tends to deform into a rhombic dodecahedron. Consequently, the silica nanoparticles may be restricted to filling in the gap between each polymer dodecahedron with one layer only. Thus, the ϕ_c_ in such a restricted network could be remarkably decreased down to ∼0.13 (*calculation provided* in the Supporting Information). Furthermore, this critical volume fraction may highly depend on the supracolloid polymer–silica size ratio, with a smaller size ratio leading to a higher critical volume fraction. The *G*′_r_ predicted using ϕ_c_ = 0.13 as boundary condition (Figure S10) indicated indeed much better match with the experimental data, as compared to *G*′_r_ predicted with ϕ_c, eff_ = 0.30. Unfortunately, the small number of nanocomposite coating samples available for this study may not be representative enough to make a proper fit to the mechanical models mentioned. However, it seems reasonable to assume that the critical volume fraction of the CB-Si nanocomposite coatings is expected to be below or just around ϕ_Si_ = 0.125. and a *further discussion is provided in* the Supporting Information.

The relative Young’s modulus (*E*_r_) measured for the CB-Si and PA-Si coatings was also analyzed (Figure 4b). The Guth–Gold equation^75^ can be used to estimate the relative Young modulus (*E*_r_) of the nanocomposite coatings, given by
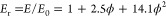
3where *E* is the Young’s modulus measured for the nanocomposite coating and *E*_0_ is the Young’s modulus measured for a coating with polymer only. In equation 3, only the effect of the hydrodynamic interaction between “fillers beads” (colloidal) pairs are considered. However, for more concentrated systems (ϕ_Si_ > 0.1), additional factors will contribute significantly to the elastic modulus of the composites, such as the aggregation of the fillers and the presence of a percolated network.^75−78^ Also, in this case, a good fit of our data to predicting models may be unfair due to the low number of coatings available, but it seems reasonable to assume that the trend observed for the *E*_r_ of the CB-Si coatings in Figure 4b (*stars*) (of exponential increase with ϕ_Si_) is related with these contributing factors, provided by the homogeneous distribution of the silica nanoparticles throughout the CB-Si coatings (Figure 3) and creation of an interconnected nanonetwork. A *further discussion is provided* in the Supporting Information. The *E*_r_ of the PA-Si nanocomposite coatings, however, “seems” to indicate a “near-linear” relation with the increase of the silica volume fraction, Figure 4b (*circles*). The PA-Si coatings have a heterogeneous structure and a gradient of silica distribution, with silica segregation at the air–coating interface (Figure 3). Hence, the measured modulus could most likely be an average result of the presence of silica networks with different densities.

The tensile stress (ε)—strain (σ) curves of the nanocomposite coatings were also recorded and are shown in Figure 4c,d. All the coatings showed a surprisingly high deformation. Even CB-Si.25, which has a volume fraction of silica approximate to an estimated critical value (ϕ_c_ = 0.125), showed good stretchability without breaking at least until 400% strain (limit of the testing device used). Previous studies with filled polymer rubber systems showed that the composite tends to become brittle above the (critical) percolated volume fraction, even in well-dispersed systems that use polymer particles as templates.^10,13^ Although the yield stress point cannot be accurately determined for all nanocomposite coatings, a clear trend of decreasing yield stress point with increasing silica content can be identified for both CB-Si and PA-Si coatings (estimations in Figure 4c,d). Furthermore, some other observations can be highlighted. When CB-Si coatings are in the plastic region, between ∼50–300% strain, the strain hardening is significantly larger for films with the higher silica mass percentage. Above 300% strain, the strain hardening of CB-Si.15 and of the polymer coating EDT-L.33 continues to increase linearly, but this was not the case for CB-Si.20 and Si.25. A plausible explanation would be that a higher content of S–S bonds restricts the mobility of the polymer chains. On the other hand, it could also be that the increased silica network density of the coatings with the higher silica fraction disturbs the polymer entanglements, especially at the polymer particles interfaces, lowering their viscosity in the large deformation region. For PA-Si films, the strain hardening is also higher for films with higher silica content but shows a slope change beyond 300% strain at all fillers mass fractions, most likely due to the absence of the restricting S–S bonds. Furthermore, the strain hardening in the plastic region (50–200% strain) of the PA-Si coatings was lower than the one of CB-Si coatings with the same silica volume fraction. This might suggest that in these nanocomposite coatings, the movement of the polymer chains between the silica percolating networks largely dominates in the plastic region.

To further confirm our interpretations on *E* – ϕ_Si_ and *G*’ – ϕ_Si_, PA-Si.40 films were also measured (Table S1). The stress–strain curve of PA-Si.40 is given in Figure S3. The storage modulus of the nanocomposite coatings with a significantly larger silica mass percentage (40 wt %) was very close to that of CB-Si.25. This indicates that the S–S bonds are not the only responsible factor for the strain hardening, but a high silica mass percentage in general, i.e., the presence of dense silica networks, has a strong impact on the strain hardening properties. Accordingly, the Young’s modulus of PA-Si.40 was more than twice the value measured for PA-Si.25.

### Nontriggered (Viscoelastic) Recovery of Blunt Surface Scratches

**Figure 5:**
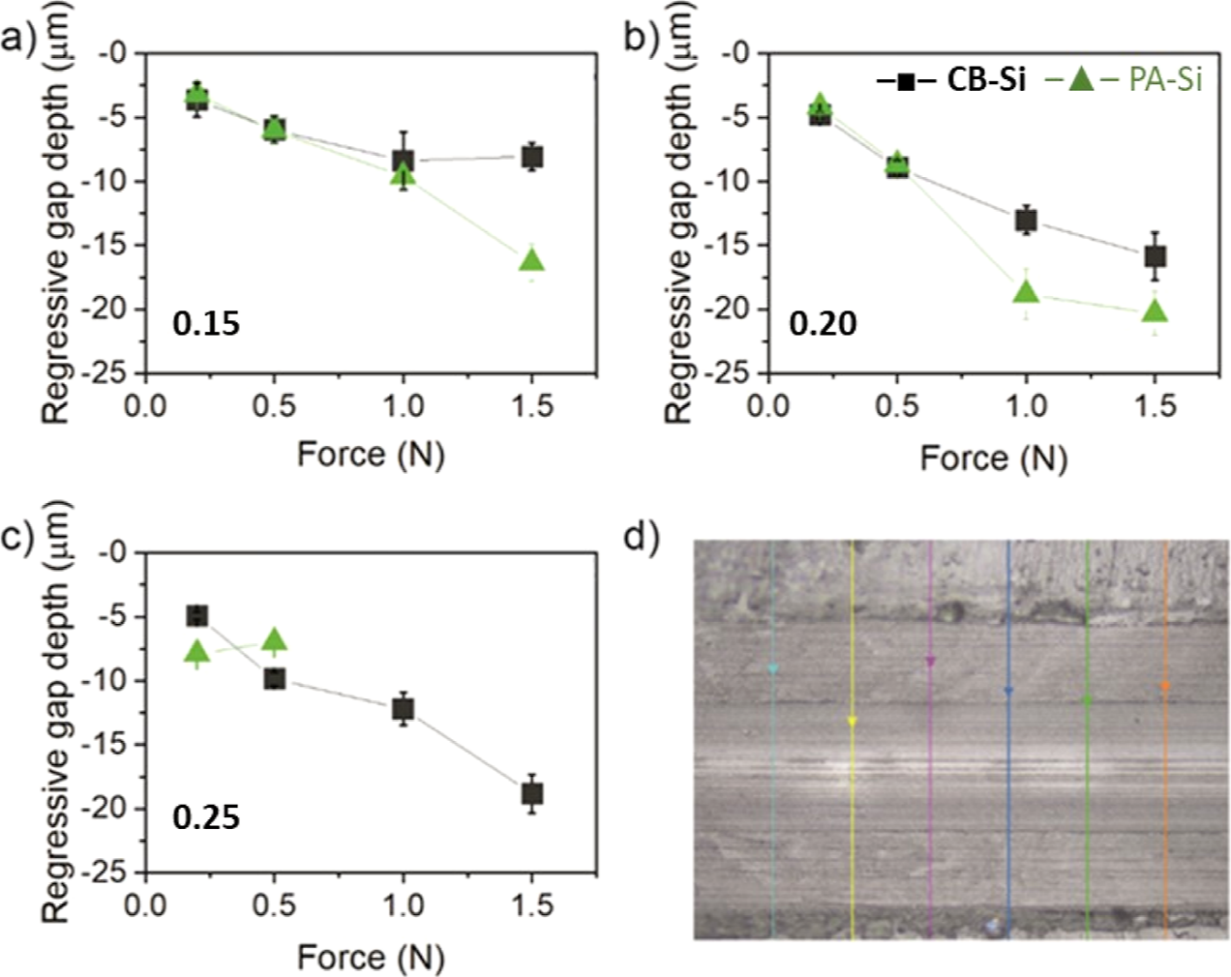
Regressive gap depth of scratches made on nanocomposite coatings, CB-Si black (■) and PA-Si green (▲) with (a) 15, (b) 20, and (c) 25 wt % silica weight fractions. (d) Optical microscopy image of a scratch. The 6 solid lines in the image indicate the gap depth measuring positions and direction.

The nontriggered recovery of the coatings from surface physical damage was examined by inflicting controlled (surface) scratches and evaluating the recovery immediately after the damage, at room temperature and in the absence of any specific trigger (i.e., no photoirradiation). An MST equipped with a Rockwell tip was used to perform scratches with a controlled load force on the air–coating interface of the nanocomposite coatings. The original penetration depth of scratches made with different load forces was first studied in detail for CB-Si.15 and PA-Si.15 to establish the optimal scratch settings (Figure S4). Immediately after the damage, the scratch recovery was monitored by measuring the “regressive” gap depth with a confocal microscope (Figure 5d). A small regressive gap depth value (closer to 0) in Figure 5 means a better recovery of the original scratch, i.e., better immediate healing. For the sake of reference, coatings with polymer particles only L.33 and EDT-L.33 were also tested. Nevertheless, these coatings failed across the polymer network already at a 0.5 N load force and could therefore not be represented here.

As seen in Figure 5, no full recovery of the penetration gap depth was achieved for any of the coatings or load forces tested (i.e., all values differ from zero). Furthermore, the surface scratch recovery was generally weaker for coatings with a higher silica mass fraction. For coatings with the highest silica fraction and absence of dynamic bonds (PA-Si.25), a large fishbone-like fracture was observed (Figure S5), and the quantification of the regressive gap depth (beyond 0.5 N) became impossible (Figure 5d), which are clear signs of a weaker cohesive coating strength. However, for the coatings with dynamic bonds and the same high silica content (CB-Si.25), a higher local deformation without coating cohesive failure was observed (Figure 5d). The presence of dynamic bonds seems to provide a sort of surface damping effect (energy dissipation), which highlights the benefit of dynamic bonds as an approach to increase scratch resistance in coatings with high silica particles content. For CB-Si.15 and CB-Si.20, the surface scratch recovery at high scratch loads was higher than that for their PA counterparts. The maximum recovery was found at 1.5 N scratch load for the CB-Si.15 coatings (with an initial penetration depth of ∼80 μm, Figure 5a), which showed a remarkable surface recovery up to a final residual gap depth of ∼8 μm, Figure 5a. This seems to indicate that the CB coatings release the stored entropic energy (related to polymer chains conformations) in a more efficient manner, presumably due to the presence of the dynamic bonds, a process found to occur within 1 min under the proper conditions even for larger surface cuts.^68^ As a note, with the performed experiments, we may not be able to distinguish any contribution by surface plasticity effects. Considering also the very different compositions of the coating surfaces (in terms of silica/polymer concentration), a more surface dedicated technique, which could assess at a much smaller (molecular) scale recovery, would be required to account for such contributions.

The scratch tests show how the presence of silica increases the coating’s scratch resistance. As indicated above, coatings with polymer particles only, L.33 and EDT-L.33, failed already at 0.5 N load force and could therefore not be tested. Concurrently, surface scratch recovery is weaker for coatings with the highest silica content. The hardness of nanocomposites can be generally correlated to the presence of nanofillers;^79^ however, in these nanocomposite coatings, the particular surface structure, such as polymer and/or silica domains, may also play a vital role in the regressive gap depth, i.e., on recovering from surface scratches. As shown in Figure 3, the concentration of silica nanoparticles (*light gray smaller spheres*) at the air–coating interface increases with the silica mass fraction, which will certainly influence the scratch recovery. Due to the stratification of silica on the PA-Si coatings, the number of silica nanoparticles at the air interface of these coatings was much higher than for CB-Si with an equivalent silica content.^58^ A high silica content leads to a high brittleness of the coatings. This could explain the bigger failure of the PA-Si coatings than of the CB-Si coatings as well as the larger regressive gap depths observed for scratches made with higher load forces (up to 1.5 N).

### UV-Triggered Recovery of Sharp Surface Cuts

**Figure 6:**
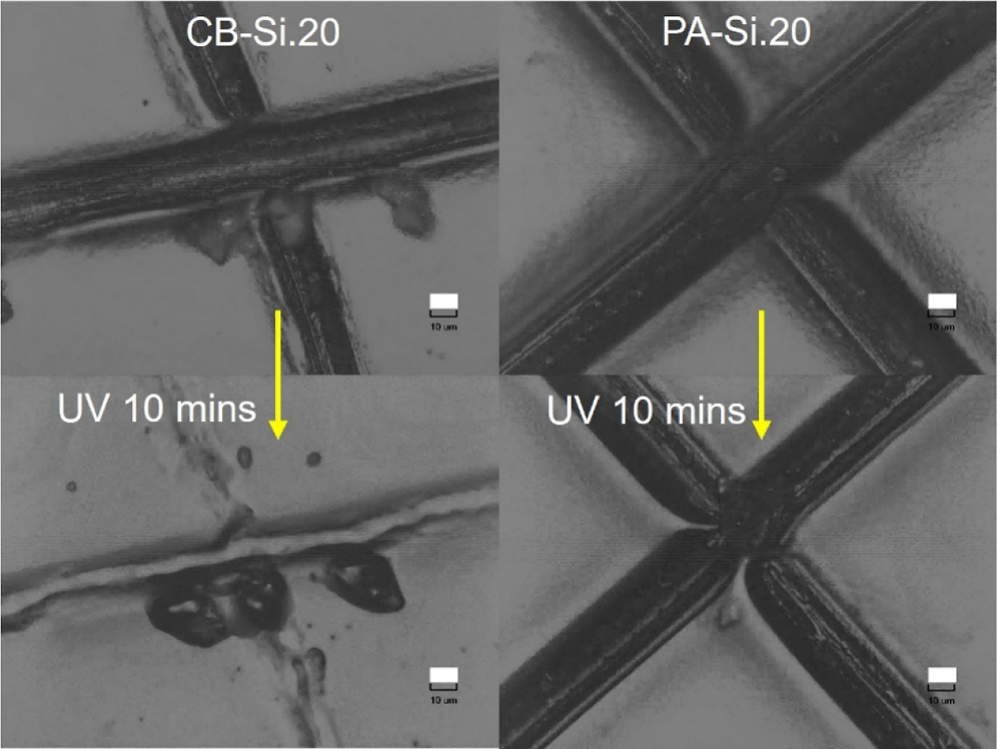
Optical microscopy images of nanocomposite coatings with silica and disulfides (CB-Si.20) and without disulfides (PA-Si.20): *top*—blade notch cuts and *bottom*—blade notch cuts after 10 min of UV irradiation (scale bars = 10 μm).

The ability of the reversible disulfide bonds to accelerate healing of surface cuts upon UV irradiation was further explored for the best coatings with disulfides (CB-Si.20 and CB-Si.15) and without disulfides, PA-Si.20 and PA-Si-15. The coatings were damaged using a sharp surgeon scalpel blade at room temperature, and a cross-notch cut was applied as shown in Figure 6. The difference in the appearance of the cross-notch is attributed to the lack of control of the blade-cut or the different surface properties of the coatings. The cut coatings were irradiated for 10 min with UV radiation (320–500 nm) and inspected immediately after the damage with an optical microscope. The depth (or the volume) of the notch cuts was difficult to quantify with optical microscopy, but the healing and closure of the notch cuts could be qualitatively (visually) evaluated, Figure 6.

Both CB-Si.20 and CB-Si.15 nanocomposite coatings showed a clear change (recovery) of the notch cut after UV irradiation (bottom image, Figure 6*left*). Conversely, the notch cut made on the PA-Si.20 coating presented only a negligible difference (Figure 6, *right*). Hence, it is reasonable to assume that the healing and partial closure of the notch cut observed for the CB-Si coatings can be attributed to the presence of reversible disulfide bonds. The short duration of the UV illumination and the absence of detectable healing when a coating without reversible bonds (PA) is irradiated in the same conditions indicate that any potential recovery due to local heating during the UV irradiation is negligible.

For the CB-Si coatings, the opening of the disulfide bonds triggered by the UV irradiation seems to allow viscous flow and further assist the cut closure. It has been widely reported that viscoelastic flow (entropy release related to different conformations of the polymer chains in the coatings network) promotes lateral displacement leading to interfacial contact, a very important step for the damage (gap) closure.^68,80^ Concurrently, although the PA-Si coatings showed a certain degree of viscoelastic (nontriggered) recovery of surface scratches (Figure 4), this was obviously not enough to significantly change the cut notch appearance in Figure 6. What is also notable is that for CB-Si.25, no obvious recovery of the notch cuts was observed after UV irradiation (Figure S6). This result seems to indicate that above the critical silica volume fraction, the polymer mobility is overall significantly reduced, as discussed in the previous section on mechanical properties, and not even a possible reshuffling of the disulfide bonds is sufficient to promote viscous flow and interfacial contact and, thus, damage recovery.

To further investigate the healing behavior, surface scratches inflicted in the same controlled manner as described in the previous section (with an indenter and variable load force) were made and immediately irradiated with UV for 10 min. The coatings were UV irradiated immediately after the surface damage, and the regressive gap depth was measured immediately after. The results (reported in Figure S7) showed that the UV irradiation had a minimal effect on the surface recovery of the blunt surface scratches of the CB-Si coatings, compared to the immediate recovery right after damage and before irradiation. Hence, while the breaking of the disulfide bonds at the polymer–silica surfaces may promote viscous flow and contact at the broken interfaces, which is essential to recovering (at least partially) deeper cut damage, it seems to have a negligible impact on the recovery of shallower surface scratches. We relate this to the difference between lateral compression during cut damages and downward compression during surface deformation leading to differences in entropy storage (related to the conformation of the polymer chains in the coatings network) and therefore different displacements during healing, a factor so far not studied in the field or autonomous polymer healing. It could also be the case that the surface scratch recovery will still slightly increase over time, but this was not evaluated in our work (the regressive gap was only measured immediately after UV irradiation).

### Effect of Disulfide Content on the Interfacial Healing of Cuts

**Figure 7:**
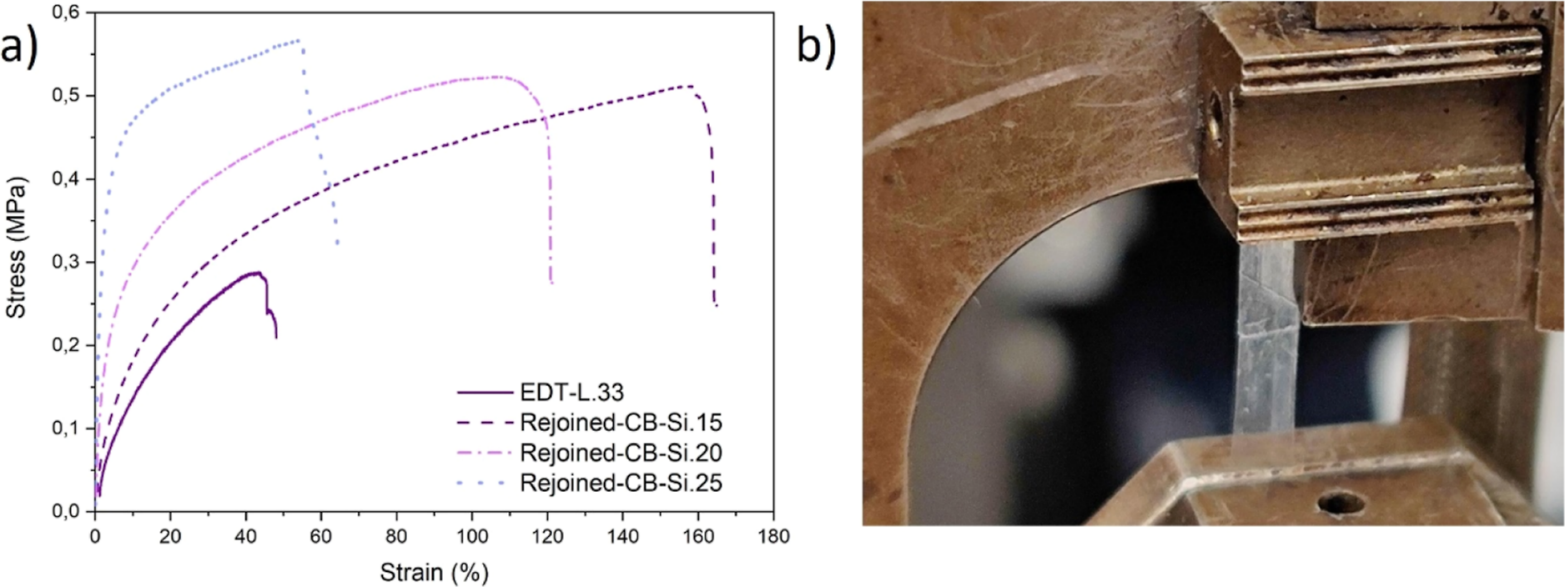
Mechanical properties of healed nanocomposite coatings—(a) stress–strain curves for rejoined pieces of CB-Si and EDT-L.33 (modified-polymer) and (b) digital photograph of rejoined and stretched CB-Si.15 nanocomposite coating specimens during the tensile test.

To study the effect of the disulfide bonds on the recovery of the coatings’ mechanical properties, free-standing films were damaged (cut in two), subjected to healing (the two parts were aligned, brought in contact, and UV-irradiated), and finally analyzed with tensile tests (Figure 7b).

As expected, for PA-Si.20, the two pieces remained split after UV irradiation, while the CB-Si.20 pieces rejoined and stayed as one piece, as qualitatively evaluated with a gentle manual stretching. These results provide further evidence that UV irradiation did not create a local temperature increase, which would be the main cause of the healing process (i.e., PA-Si20 cuts did not reattach). It further confirms that UV activation of the disulfide bonds is indeed the mainly responsible driving force for the interfacial recovery of CB-Si.20 (displacement and interfacial restoration). To obtain a quantitative estimation of the role of the disulfide bonds and silica content on interfacial healing, all the CB-Si nanocomposite coatings, as well as the EDT-L.33 polymer coating, were tested with the same cut–rejoin method, followed by mechanical analysis in the tensile mode of DMA.

The stress–strain curves of the healed parts and the healing efficiency of the Young’s moduli are shown in Figure 7a and Table 2, respectively. Compared to the original films shown in Figure 4c, the healed samples (Figure 7a) show lower elongation, yield stress, and stress at break for all samples. However, the trends observed for the strain hardening and yield stress were the same (higher for films with higher silica content). Comparatively, the EDT-L.33 coating fractured at the lowest elongation and stress, Figure 7a, which clearly indicates that the reinforcement effect of the coatings with silica nanoparticles (and networks) was beneficial to maintaining the mechanical properties of the rejoined pieces, as observed also during the blunt scratch testing.

**Table 2:** Characterization CB-Si Nanocomposite Films before and after the Healing Process (Cut–Rejoined Pieces + UV Irradiation)—Young’s Moduli of Original Coatings (*E*), Young’s Moduli of Healed Coatings (*E*_h_), the Healing Efficiency of the Young’s Moduli (*H*_E_), and Strain at Break (σ_b_) and Stress at Break (ε_b_) of the Healed Coatings

*C*_Si_ (wt %)	*E* (MPa)	*E*_h_ (MPa)	*H*_E_ (%)	σ_b_ (%)	ε_b_ (MPa)
0	0.7 ± 0.2	2.0 ± 0.1	265^b^	59 ± 19	0.33 ± 0.05
15	2.6 ± 0.2	2.8 ± 0.2	108^b^	183 ± 25	0.53 ± 0.03
20	4.8 ± 0.9	5.7 ± 0.2	120^b^	114 ± 10	0.54 ± 0.03
25	12.3 ± 0.1	10.8 ± 1.3	87	56 ± 3	0.57 ± 0.03
20^a^	4.8 ± 0.9	11.0 ± 1.8	229^b^	69 ± 32	0.60 ± 0.21

a Measured 20 days after.

b Value overestimated due to restructuring of the cut–rejoined coatings (interface) structure.

Finally, the rejoined films consistently show a significantly higher Young’s moduli (*E*_h_) than the nondamaged ones, except for CB-Si.25, Table 2. A possible reason for this observation could be the polymer–particle structure reorganization under UV irradiation and/or with time, which has not been studied in detail in this work. Although only about 2 mm (of the total 5 mm entry length of the tensile sample) was exposed to the UV irradiation, it is worth pointing out that two cut pieces were put in contact at their cross-sections without being submitted to compression forces (rejoined by clamp pressure only) and without overlapping the two pieces. Other researchers have also reported an increase in Young’s moduli after healing two previously cut coatings species, but the measured specimens were usually reprocessed at high compression forces and/or temperature.^43^ Not surprisingly, the CB-Si.25 film showed the lowest healing efficiency as the high load of silica nanoparticles may be in this case restricting the polymer mobility at the interface, as also observed for the blunt-notch cuts.

To evaluate possible time effect on the healing process, the CB-Si.20 cut, rejoined, and UV-irradiated films were stabilized in ambient conditions for 20 days (last entry, Table 2). The E modulus of this film was much higher than that of the nondamaged films. These results indicate indeed that the polymer–composite homogenization persists for a long time scale and that there is an entropy-driven rearrangement to the most favorable conformation of the polymer chains in the coatings network, once the initial interfacial contact has happened upon opening of the disulfide bonds.

Even if the increase in Young’s moduli after the healing procedure on rejoined pieces suggests polymer–particles film restructuring, the clear interfacial recovery in all CB nanocomposite films and absence of any interfacial restoration in the PA systems offer a final clear evidence of the role of the reshuffling disulfide bonds in the healing process in nanocomposite supracolloidal water-borne coatings.

## Conclusions

Water-borne nanocomposite coatings containing reversible disulfide bonds were prepared from supracolloidal aqueous dispersions. The supracolloids were obtained by simply mixing thiol-functionalized polymer particles and disulfide-functionalized silica nanoparticle dispersions. A disulfide cross-linker was added to the supracolloidal dispersions, which were further applied on a substrate and dried in ambient conditions to obtain cross-linked coatings in which the silica and polymer particles were covalently bound (CB) via disulfide bridges. In these coatings, the silica nanoparticles create a 3D nanonetwork, which ensures their homogeneous distribution throughout the cross-section and avoids their segregation/agglomeration at the interfaces. This well-defined silica percolating network lowers the critical volume fraction of the nanocomposite coatings to around 0.13, compared to that of other well-dispersed nanocomposite systems (0.30).^73^ This low critical volume fraction influences the viscoelastic properties (and recovery) of the coatings.

The storage (*G*′) and Young’s (*E*) moduli of the covalently bound nanocomposite coatings significantly increased with the increase of silica content; up to more than 30-fold (for *G*′) and 15-fold (for *E*), when compared to the values of the polymer films without fillers. Conversely, the CB coatings with the lowest silica content (15%) showed the best nontriggered (viscoelastic) recovery and surface healing properties upon both blunt scratches and sharp razor blade cuts. When the polymers were cut in two and further UV-irradiated when in contact, a clear interfacial recovery was measured. Nevertheless, the posthealed nanocomposite coatings also showed a significant change in properties (i.e., increase of *E*), which deserves further attention in future studies.

Overall, the incorporation of reversible cross-links via −S–S– bonds at the interfaces of the polymer–silica supracolloids considerably improved the mechanical properties and damage recovery capability of the nanocomposite coatings and shows high potential for water-borne healable coatings. This work provides a fresh perspective for the control of the distribution of nanofillers in water-borne coatings with an extended lifetime, provided by (physical) damage healing capabilities upon use of readily available triggers, such as UV irradiation.

## Supporting Information Available

Estimation of the critical volume fraction of silica in nanocomposite coatings, Raman spectra and consideration on thiol–disulfide exchange on the nanocomposite coatings upon annealing; additional loss moduli of nanocomposite coatings; discussion on potential modelling of the mechanical properties of the nanocomposite coatings, and additional regressive gap depth measurements from scratch tests (PDF)
