# Supplementary material for: Healable Supracolloidal Nanocomposite Water-Borne Coatings
Source: ACS Appl Polym Mater. 2024 Jul 22;6(15):8830–41. doi: 10.1021/acsapm.4c00946 (PMC11320382; doi:10.1021/acsapm.4c00946)
Supplement: Supplementary file 1 — ap4c00946_si_001.pdf [file ap4c00946_si_001.pdf]

SUPPORTING INFORMATION

Healable supracolloidal nanocomposite water-borne coatings

Siyu Li[a], Leendert G. J. van der Ven[a], Santiago J. Garcia[b], A. Catarina C. Esteves[a,c]

[a] *Laboratory of Physical Chemistry, Department of Chemical Engineering and Chemistry, Eindhoven University of Technology, P.O. Box 513, 5600 MB, Eindhoven, The Netherlands.*

[b] *Aerospace Structures and Materials Department, Faculty of Aerospace Engineering, Delft University of Technology, Kluyverweg1, 2629 HS, Delft, The Netherlands*

[c] *Institute for Complex Molecular Systems (ICMS), Eindhoven University of Technology, P.O. Box 513, 5600 MB, Eindhoven, The Netherlands*

* Corresponding author: a.c.c.esteves@tue.nl

SUPPORTING FIGURES

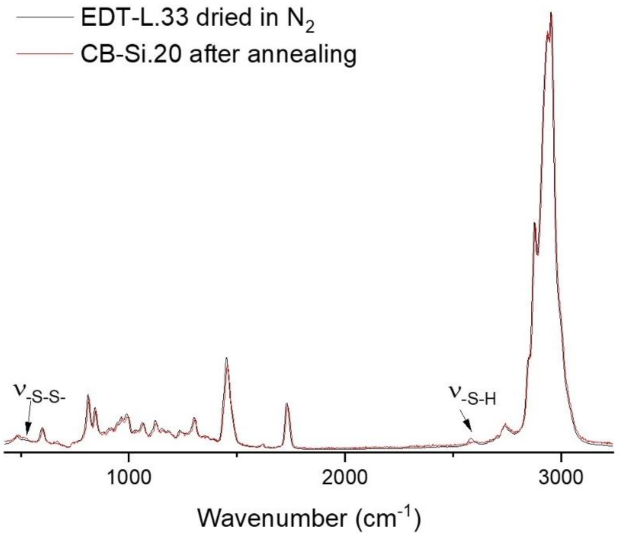

Figure S1: Raman spectra of: **a)** EDT-L.33 polymer coating dried in N_2 and **b)** CB-Si.20 nanocomposite after annealing at 70 °C.

Figure S1 illustrates the oxidation of thiol groups after the drying of coatings in the air. The EDT-L.33 coating was dried in an N_2 atmosphere to avoid the oxidation of thiol groups, and then subsequently examined by Raman microscopy. On the contrary, the CB-Si.20 coating was measured after the coating was dried in air and annealed for 48 h. The $\upsilon_{\text{-S-S-}}$ overlapped with $\upsilon_{\text{-Si-O-}}$ in CB-Si.20 coating so that the higher signal observed at 500 cm^{-1} was possibly attributed to the silica nanoparticles in the coating instead of the formation of disulfide groups. More importantly, $\upsilon_{\text{-S-H}}$ at 2570 cm^{-1} of the CB-G2.4-Si.20 coating had a significantly lower signal compared to unoxidized polymer coating, which indicated that the majority of thiol groups were oxidized by air and converted to disulfide groups. The conversion of thiol groups was estimated to be around 80% which was obtained by comparing the different areas of the $\upsilon_{\text{-S-H}}$ band from both samples.

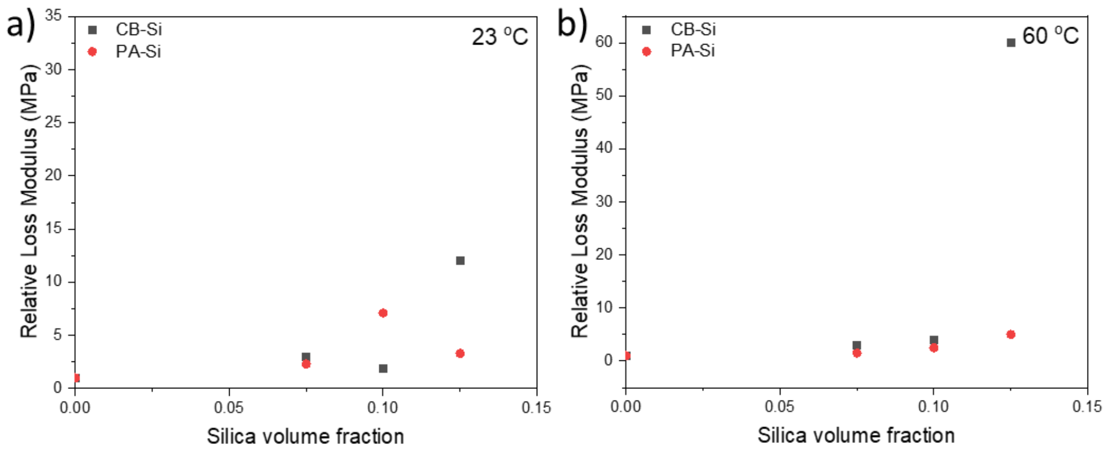

Figure S2: The relative loss moduli of nanocomposite coatings with different silica volume fractions at (a) 23 °C and (b) 60 °C.

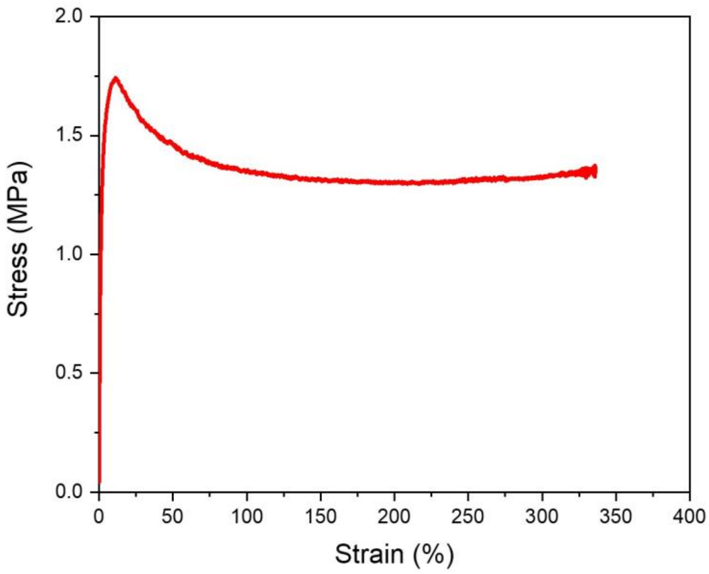

Figure S3: Stress-strain curve of the PA-Si.40 nanocomposite coating.

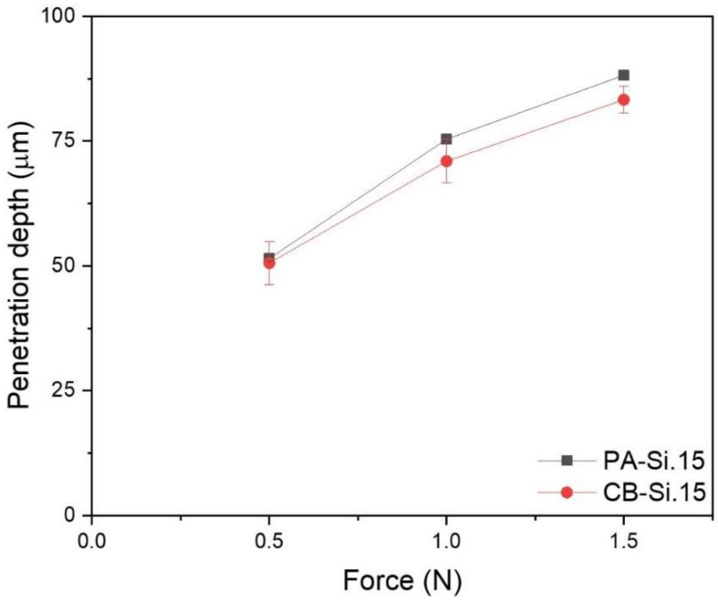

Figure S4: Penetration depth-loaded force curves for the indentation of CB-Si.15 and PA-Si.15 nanocomposite coatings.

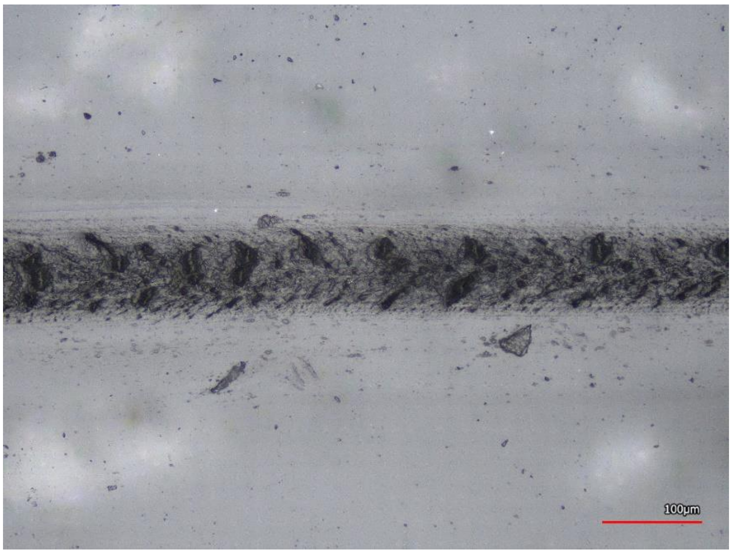

Figure S5: Optical microscope image of the scratching on PA-Si.25 coating using 1.0 N loaded force (scale bar = 100 μm)

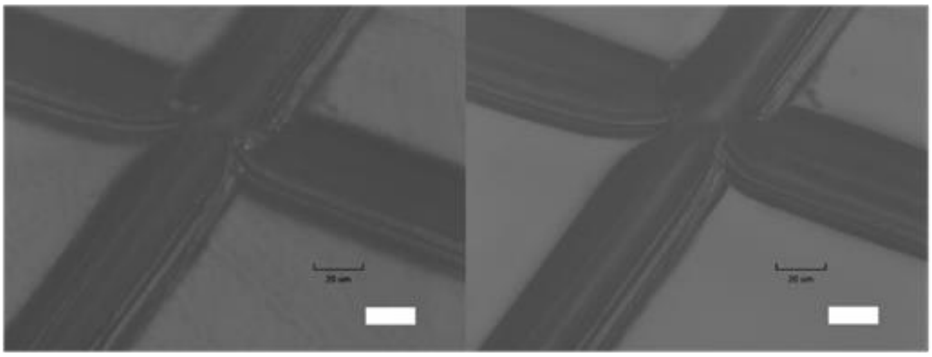

Figure S6: Optical microscope images of the notches on CB-Si. 25 coatings made by razor blade (*left*) and the notches after 10 minutes UV irradiation (*right*) (Scale bars = 20 μm).

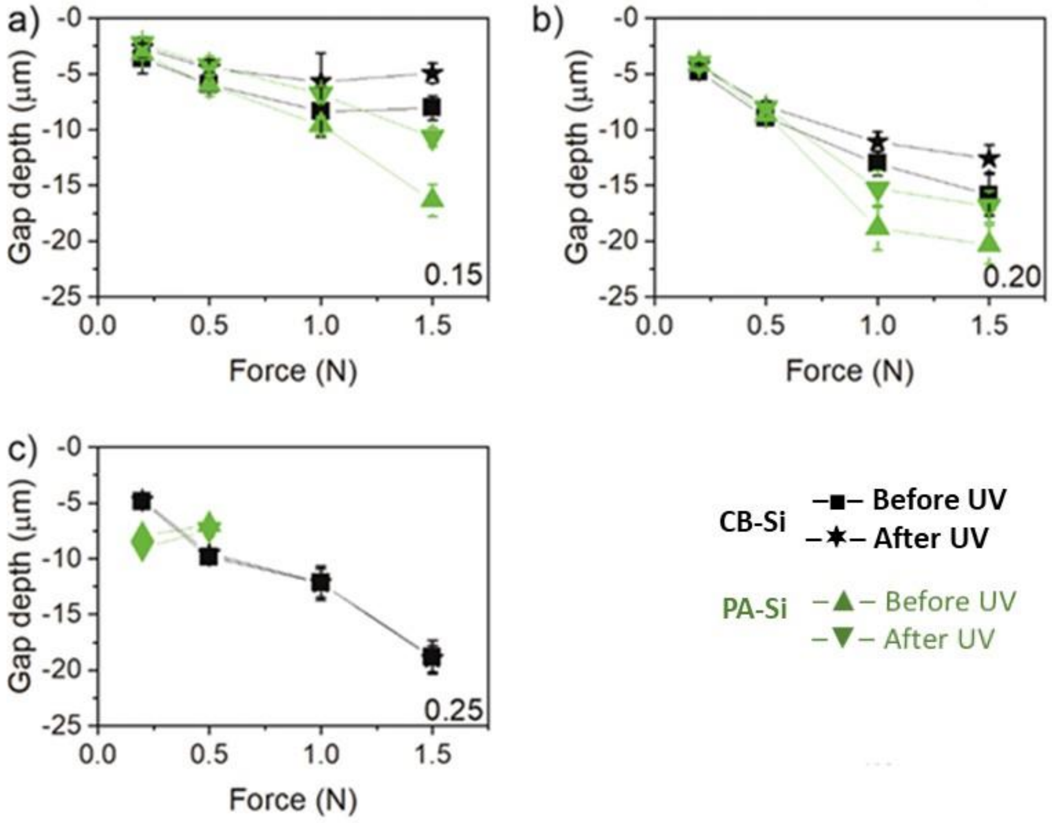

Figure S7: Regressive gap depth of scratches on nanocomposite coatings, before and after 10 minutes UV radiation *versus* loaded force applied to make the scratch, with silica mass fraction of: **(a)** 15 wt%, **(b)** 20 wt% and **(c)** 25 wt%.

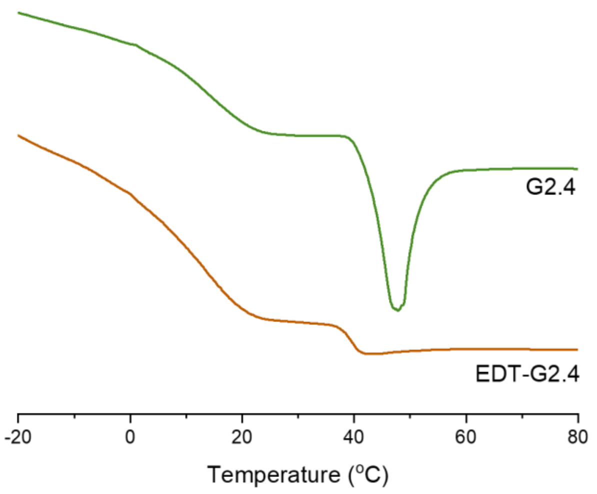

Figure S8: Differential Scanning Calorimetry (DSC) of the two polymer films

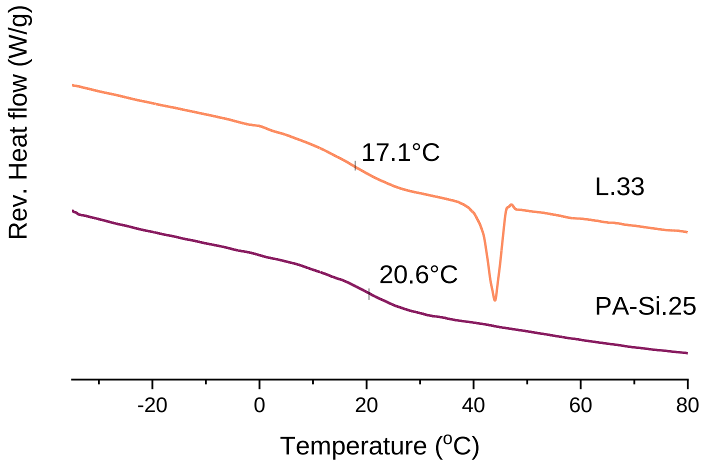

Figure S9: Reverse Heat Flow of Modulated Differential Scanning Calorimetry (DSC) of polymer film L.33 and a supracolloidal film PA-Si.25.

SUPPORTING DISCUSSION

Estimation of critical volume fraction:

As the rhombic dodecahedron and spherical polymer particles were equal and the volume of the rhombic dodecahedron can be calculated as

$$V = \frac{16\sqrt{3}}{9} a^3 \ , \qquad \text{[S1]}$$

where a is the length of the sides. Then the ratio of $a/r \approx 2.035$ can be obtained, where r is the radius of the polymer core. As Scheme S1 showed, the critical volume fraction of silica can be estimated as

$$\phi_c = 0.64(V_s - V_p)/V_s \ , \qquad \text{[S2]}$$

where V_s is the volume of the dodecahedron with the side of a_s and V_p is the volume of the polymer core.

$$a_s = a + \frac{\sqrt{(\sqrt{5}+1)^2+4}}{\sqrt{5}+1} r_s \approx a + 1.18 r_s \ . \qquad \text{[S3]}$$

Hence, as the r was 128 nm and r_s was 27 nm, the ϕ_c was approximately 0.13.

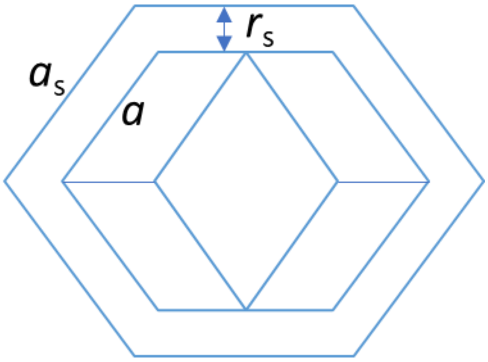

Scheme S1: The projection of rhombic dodecahedron.

Discussion on potential modelling of the mechanical properties of the nanocomposite coatings:

From the data in Figure S10, it can be noticed that for G'_r of data measured at 23 ℃ (Figure S10 a, *squares*) the film was in the glass-rubber transition regime, were the Poisson's ratio can vary significantly, but the G'_r data obtained at 60 ℃ (Figure S10 a, *triangles*) further confirms a good match with the estimation considering ϕ_c= 0.13. Besides, the relative loss moduli G''_r of the nanocomposite coatings ($G''_{nanocomposite}$ / $G''_{polymer}$, loss modulus of a nanocomposite film over that of film with polymer only) also indicates a good match of the measured data to an estimated ϕ_c = 0.13, independently of the measuring temperature (Figure S10). Hence, overall, it seems reasonable to assume that the CB-Si.25 (ϕ = 0.125) nanocomposite coatings are very close to having the ϕ_c content (*i.e.* estimated at 0.13).

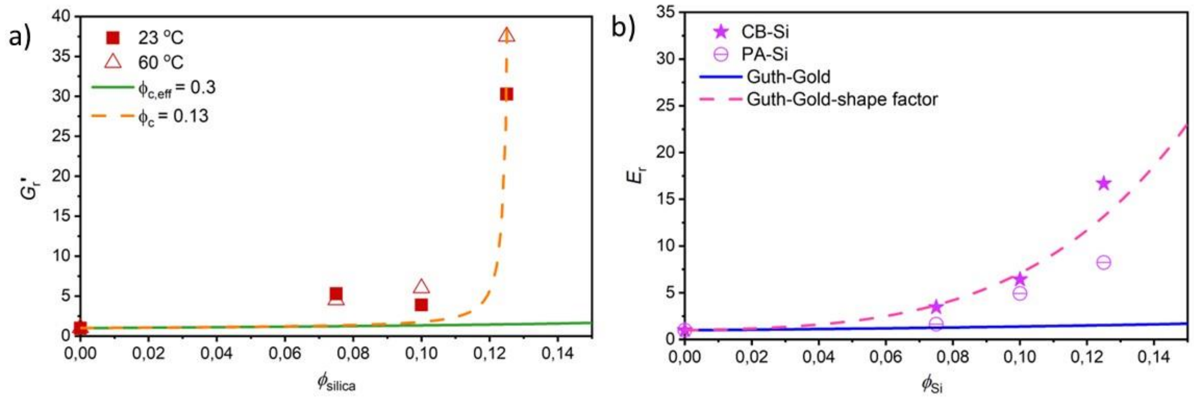

Figure S10: Viscoelastic properties of nanocomposite coatings: (a) Relative storage moduli (G'_r) *versus* ϕ_{Si} of CB-Si at 23 ℃ and 60 ℃, measured at 1 rad/s angular frequency and 0.05 % strain (*orange symbols*). Lines represent G'_r - ϕ_{Si} relation calculated by the Kerner equation, with $\phi_{c,eff}$ = 0.3 (*solid green*) and with $\phi_{c,eff}$ = 0.13 (*dashed orange*). **(b)** Relative Young's moduli (E_r) *versus* ϕ_{Si} of CB-Si and PA-Si measured at 23 ℃ (*pink symbols*). Lines represent the E_r - ϕ_{Si} relation calculated by the Guth-Gold equation (*solid blue*) and with shape factor modification (*dashed pink*).

In relation to the E_r of the nanocomposite coatings, Guth proposed to modify the volume fraction with a shape factor f (= length/width) where 'glassy bridges' link the "fillers beads" as "rod-like fillers". The adapted E_r is then given by,

$$E_r = 1 + 0.67(f\phi) + 1.62(f\phi)^2, \quad [S4]$$

where $f\phi$ is the effective volume fraction of fillers. Moreover, for more concentrated systems, *e.g.*, with percolating filler networks, it is also known that the relative viscosity(/modulus) can be expressed as,

$$\eta_r = 1 + k_1\phi + k_2\phi^2 + k_3\phi^3 + k_4\phi^4 , \qquad [S5]$$

where k_1=2.5 (Einstein) and k_2=14.1(Guth-Gold).[77] The value of k_3 and k_4, which are often also much higher than k_2, varies from the different experimental systems due to the formation of agglomerations and the hydrodynamic effect.[78,79] Therefore, to obtain a better fitting of E_r and ϕ for CB nanocomposite coatings, an analogy of equation [s2] was used with a higher order of $f\phi$

$$E_r = 1 + 0.6(f\phi) + 1.6(f\phi)^2 + 2.6(f\phi)^3 + 3.6(f\phi)^4 , \qquad [S6]$$

where $f \approx 9$ was estimated from the ratio between the diameters of the polymer core and corona silica nanoparticles of the supracolloids (Figure S10 b, *solid blue line*). The pre-factors 2.6 and 3.6 were chosen under the assumption of a trend from first (0.6) to second (1.6), to third and fourth pre-factors. We understand that this remains a rough assumption, but unfortunately, we could not find any reported values in the previous literature for the 3rd and 4th pre-factors.

Hence, the application of Equation [S6] to estimate E_r describes more accurately the experimental data obtained for the CB-Si nanocomposite coating (Figure 4 b, *stars*) as compared to Equation [3] in main paper (*dashed pink line*), since it takes into consideration the significant contribution of the fillers percolated network to the elastic modulus. For the PA-Si coatings, the results strongly deviate from any of the simple model's predictions, as the E_r-ϕ_{Si} curve is expected to be a complex modulus influenced by a percolating silica network with a gradient of silica aggregation.

References from main paper:

(76) Hughes, A. J. The Einstein Relation between Relative Viscosity and Volume Concentration of Suspensions of Spheres. Nature 1954, 173 (4414), 1089–1090.

(77) Thomas, D. G. Transport Characteristics of Suspension: VIII. A Note on the Viscosity of Newtonian Suspensions of Uniform Spherical Particles. J Colloid Sci 1965, 20 (3), 267–277.

(78) De Kruif, C. G.; Van Iersel, E. M. F.; Vrij, A.; De Kruif, G.; Van Lersel, E. M. F.; Russel, W. B. Shear Thickening, Frictionless and Frictional Rheologies in Non-Brownian Suspensions. Transactions of the Society of Rheology 1985, 83, 1693.

SUPPORTING TABLES

Table S1: The storage moduli (G'), loss moduli (G''), and Young's moduli (E) of polymer and nanocomposite films.

**Sample**	**Silica content (wt%)**	G' **(MPa)**	G_1'' **(MPa)**	G_2'' **(MPa)**	E **(MPa)**
CB-Si	0	0.11 ± 0.01	0.17 ± 0.02	0.01 ± 0.00	0.74 ± 0.21
	0.15	0.55 ± 0.08	0.52 ± 0.11	0.03 ± 0.00	2.56 ± 0.15
	0.20	0.43 ± 0.05	0.32 ± 0.04	0.04 ± 0.01	4.76 ± 0.91
	0.25	3.33 ± 0.62	2.06 ± 0.90	0.60 ± 0.40	12.34 ± 0.06
PA-Si	0	0.20 ± 0.08	0.21 ± 0.06	0.02 ± 0.02	2.27 ± 0.17
	0.15	0.49 ± 0.12	0.48 ± 0.15	0.03 ± 0.00	3.73 ± 0.38
	0.20	1.57 ± 0.38	1.49 ± 0.31	0.05 ± 0.02	11.20 ± 3.33
	0.25	1.92 ± 0.89	0.69 ± 0.39	0.10 ± 0.00	18.71 ± 0.01
	0.40	3.57	1.47	--	42.98 ± 6.38

* G', G_1'', and E measured at 23℃, G_2'' measured at 60℃, respectively.

Supporting reference

1. Li, S. *et al.* Assembly of partially covered strawberry supracolloids in dilute and concentrate aqueous dispersions. *J Colloid Interface Sci* **627**, 827–837 (2022).
